# Long noncoding RNA MIAT promotes non-small cell lung cancer progression by sponging miR-149-5p and regulating FOXM1 expression

**DOI:** 10.1186/s12935-020-01432-3

**Published:** 2020-07-28

**Authors:** Zhi Zhou, Shan Zhang, Yaqiong Xiong

**Affiliations:** Department of Respiration, The Affiliated Huaian No. 1 People’s Hospital of Nanjing Medical University, Huaian, 223300 Jiangsu China

**Keywords:** Non-small cell lung cancer, long noncoding RNA, MIAT, miR-149-5p, FOXM1

## Abstract

**Background:**

Long non-coding RNAs (lncRNAs) are a class of endogenous non-coding RNAs of longer than 200 bp that play crucial roles in cancer biology. Here, we assessed the tumorigenic properties of a long noncoding RNA, MIAT, in non-small cell lung cancer (NSCLC).

**Methods:**

Survival and clinicopathological analyses were done in a cohort of 80 patients with NSCLC. MIAT expression level were determined by real-time quantitative reverse transcriptase PCR (qRT-PCR). Dual luciferase reporter assays were employed to test the interaction between MIAT and miR-149-5p. Ectopic overexpression and shRNA-mediated knockdown of MIAT, CCK-8 and colony formation assays, Transwell migration and invasion in vitro, and in vivo tumorigenesis experiment were used to evaluate the function of MIAT.

**Results:**

MIAT was significantly up-regulated in NSCLC tissues and cell lines, and was closely associated with advanced pathological stage and poor overall survival. Gain- and loss-of-function experiments in cell lines and mouse xenograft models showed that MIAT promoted the proliferation, migration, and invasion of NSCLC cells in vitro and accelerated tumor growth in vivo. Luciferase assay, western blotting, qRT-PCR, and rescue experiments showed that, mechanistically, MIAT could directly bind to miR-149-5p, and subsequently served as a sponge to increase the expression level of Forkhead box M1 (FOXM1).

**Conclusions:**

Our study reveals that MIAT acts as an oncogene in NSCLC via a novel MIAT/miR-149/FOXM1 axis, thus providing potential biomarkers and therapeutic targets for the management of NSCLC.

## Background

Lung cancer is currently the most common cancer and the leading cause of global cancer-related mortality, and ~ 85% of all lung cancers are non-small cell lung cancer (NSCLC) [1]. Radical resection, chemotherapy, and radiotherapy are the principal treatments for NSCLC patients. However, the prognosis remains poor because the metastasis and recurrence rates are still high [2]. Hence, extensive exploration of the underlying regulatory mechanisms implicated in the progression of NSCLC is important.

Long non-coding RNAs (lncRNAs) are a class of noncoding RNAs (ncRNAs) longer than 200 nucleotides that play critical roles in a wide range of biological processes [3, 4]. In addition, dysregulation of lncRNAs has been reported to be involved in the tumorigenesis, progression, and metastasis of a variety of cancers. For instance, lncRNA UCA1 regulates PRL-3 expression by sponging miR-495 to promote the progression of gastric cancer [5]. LINC00662 promotes hepatocellular carcinoma progression via altering genomic methylation profiles [6]. Up-regulated LINC01234 promotes non-small-cell lung cancer cell metastasis by activating VAV3 and repressing BTG2 expression [7].

Myocardial infarction-associated transcript (MIAT) is first identified as a highly conserved mammalian lncRNA [8]. MIAT is involved in various cellular processes, including myocardial infarction, formation of nuclear bodies, paranoid schizophrenia and neurogenic commitment [9–12]. It has been known that MIAT directly interacts with SF1 splicing factor, therefore it is supposed to be implicated in RNA splicing and regulating gene expression. A recent study have demonstrated that promotes papillary thyroid cancer progression via sponging miR-212 [13]. Besides, upregulation of MIAT regulates LOXL2 expression by competitively binding miR-29c in clear cell renal cell carcinoma [14]. However, the potential roles and mechanisms of MIAT in NSCLC are still unclear.

In the present study, we investigated the expression pattern, biological function, and underlying mechanism of MIAT in NSCLC progression. Our data revealed that MIAT was upregulated in NSCLC cells and tissues, and correlated with poor prognosis. MIAT could interact with miR-149-5p and regulate the expression of FOXM1 to facilitate cell proliferation, migration, and invasion in NSCLC.

## Materials and methods

### Clinical samples

NSCLC tissues and matched adjacent normal lung tissues were obtained from Department of Pathology, Huaian First People’s Hospital, Nanjing Medical University. All tissues were put in liquid nitrogen and frozen immediately, and then stored at − 80 °C before RNA extraction. The present study was approved by the Nanjing Medical University Ethics Committee and was conducted according to the Declaration of Helsinki. Written informed consents were obtained from the patients.

### Cell lines and culture

The NSCLC cell lines (H1650, SPC-A1, Calu3, A549 and H1299) and immortal human lung cell line BEAS-2B were purchased from the Type Culture Collection of the Chinese Academy of Sciences (Shanghai, China). All cell lines were cultured in Dulbecco’s modified Eagle’s medium (DMEM) (Gibco, Rockford, MD, USA) supplemented with 10% fetal bovine serum (FBS), 100 U/ml penicillin, and 100 µg/ml streptomycin. The cells were cultured in a humidified atmosphere containing 5% CO_2_ at 37 °C.

### RNA extraction and quantitative real-time polymerase chain reaction (qRT-PCR)

TRIzol reagent (Thermo Fisher Scientific, Carlsbad, CA, USA) was used to extract total RNA from NSCLC cells, tissues, and normal tissues according to the manufacturer’s protocol. cDNA was synthesized from RNA using the High-Capacity cDNA Reverse Transcription kit (Applied Biosystems, Foster City, CA, USA). A SYBR Green kit (Bio-Rad Laboratories, USA) in PCR Thermal Cycler (Applied Biosystems, Foster City, CA, USA) was used to perform the qRT-PCR assay. GAPDH was used as a standard control. The relative expression was calculated by the 2^−ΔΔCt^ method.

### Western blotting analysis

NSCLC cells were extracted with RIPA lysis buffer (Beyotime, Shanghai, China). Equivalent amounts of proteins were electrophoresed by SDS-PAGE and then transferred onto PVDF membranes. Next, total proteins were incubated with primary antibodies at 4 °C overnight. Then, the membrane was washed thrice with PBS and incubated with secondary antibody. The bands were visualized using ECL-PLUS/Kit (GE Healthcare, Piscataway, NJ, USA) according to the kit instructions.

### Luciferase reporter assay

A total of 3 × 10^4^ cells were seeded into each well of the 96-well plates. The 3′-UTR sequences of MIAT or FOXM1 encompassing the miR-149-5p wild-type or mutant binding sites were synthesized. The sequences were inserted into pmirGLO luciferase reporters (Promega, Madison, WI, USA) between *Sac*l and *Sal*l restriction sites, respectively. The binding sites for miR-149-5p were mutated by Gene Mutation Kit (Takara, JAPAN) to generate the mutant MIAT or FOXM1 3′-UTR. The plasmids, including 3′-UTR of wild-type or mutant sequences from MIAT or FOXM1 and miRNA mimic were co-transfected into NSCLC cells using Lipofectamine 2000 (Invitrogen, Foster city, USA). After incubation for 48 h, a dual-luciferase reporter assay (Promega, Madison, WI, USA) was used to test the firefly and Renilla luciferase activities. Results were presented as relative luciferase activities of Renilla, which were normalized to the activity of firefly luciferase.

### shRNA, mimic, inhibitors, and lentiviral vector

Short hairpin RNA (shRNA), miRNA inhibitors, and mimic were synthesized and purified by Gene-Pharma (Shanghai, China). Lipofectamine 2000 reagent (Invitrogen, Foster city, USA) was used for the transfection. The sequences of the shRNA for targeting and silencing MIAT were as follows, sh-1: 5′-ACUUCUUCGUAUGUUCGGCTT-3′ and sh-2: 5′-GCUCUUUCCUAUUGGAUAUTT-3′. The lentivirus-mediated MIAT overexpression vector and empty vector were purchased from GeneChem (Shanghai, China).

### Cell proliferation, colony formation assay and flow cytometric analysis of the cell cycle

For cell proliferation assay, NSCLC cells transfected with sh-MIAT or MIAT-overexpression vector were seeded into 96-well plates (2 × 10^3^ cells per well). After 0, 1, 2, 3, and 4 days, 10 µl CCK8 solution was added in each well. Then, cells were incubated at 37 °C for 2 h. The absorbance of each well was measured at 450 nm using a microplate reader (Tecan, Switzerland). For the colony formation assay, the cells were cultured at 1 × 10^3^ cells per well in 6-well plates and incubated at 37 °C for 7 days. The cell colonies were fixed and stained using 4% paraformaldehyde and 0.1% crystal violet, respectively. Cell cycle analysis was implemented with PI staining by a flow cytometry (Becon Dickinson FACSCalibur, NY, USA).

### Transwell invasion assay

NSCLC cells were first seeded into the upper chamber with the Matrigel (Corning, NY, USA) for invasion assay or without Matrigel for migration assay. Cells suspended in 0.2 ml serum-free medium at densities of 1 × 10^5^ cells/well and 8 × 10^4^ cells/well for invasion and migration assays, respectively, were added to the upper chambers, and a medium supplemented with 10% FBS was added to the lower chambers. The cells were incubated for 24 h for the migration assay and 48 h for the invasion assay at 37 °C with 5% CO_2_. The cells that migrated to the lower membrane surface were fixed and stained using 4% paraformaldehyde and 1% crystal violet solution, respectively. The cells were then photographed and counted.

### Xenografts in mice

A549 cells (at a density of 5 × 10^6^ cells per mouse) with MIAT knockdown and negative control were subcutaneously injected into the upper back of BALB/c nude mice (female, 4-weeks-old). Tumor growth was monitored every week by measuring the width (W) and length (L) with calipers, and the volume (V) of the tumor was calculated using the formula V = (W^2^ × L)/2. Mice were sacrificed and examined for tumor weight after 4 weeks. The animal studies were approved by the Nanjing Medical University Animal Ethics Committee.

### Bioinformatic prediction

DIANA LncBase and TargetScan were used to compare and predict miRNAs that could directly bind to the MIAT sequence. miRNAs with high score in both DIANA LncBase and TargetScan databases were selected as the potential target of MIAT. Targetscan was used to predict the target genes of miR-149-5p. Candidates with high prediction score were selected as potential target of miR-149-5p.

### Statistical analysis

Statistical analyses were carried out using GraphPad Prism 7.0 (GraphPad Software, La Jolla, CA, USA). The statistical significance for comparisons of two groups was determined using Student’s t-test. Kaplan-Meier method with log-rank test was used to compare overall survival rates. Data are presented as the mean ± standard deviation (SD), and a two-sided *p* < 0.05 was considered statistically significant.

## Results

### MIAT was relatively overexpressed in NSCLC tissues and cell lines

**Fig. 1:**
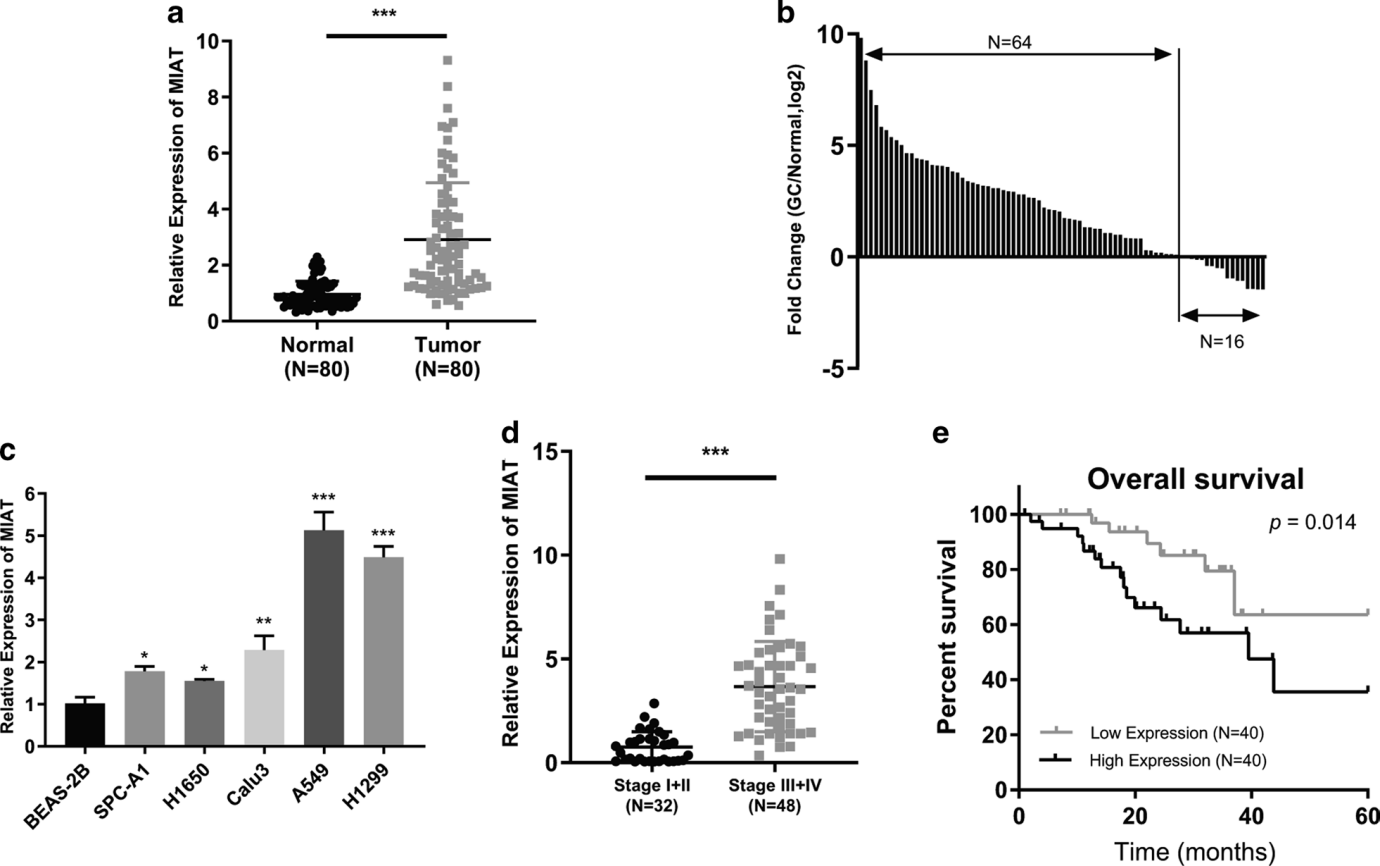
The expression and clinical significance of MIAT in NSCLC. **a** qRT-PCR analysis of MIAT in 80 paired NSCLC and adjacent normal tissues. **b** MIAT expression was upregulated in 80% (64/80) of NSCLC samples. **c** The expression of MIAT in NSCLC cell lines was detected by qRT-PCR. **d** MIAT expression levels were lower in stage I/II than in stage III/IV NSCLC. **e** Kaplan-Meier analysis indicated that patients with high MIAT expression had low survival rates. *p < 0.05, **p < 0.01, ***p < 0.001

The MIAT expression in 80 pairs of NSCLC and adjacent normal lung tissues was measured by qRT-PCR assay. The results showed that MIAT expression was considerably higher in NSCLC than in normal lung tissues (Fig. 1a). MIAT expression was upregulated in 80% (64/80) of NSCLC samples (Fig. 1b). Next, we evaluated MIAT expression in several NSCLC cell lines using qRT-PCR. MIAT demonstrated a higher expression in all five NSCLC cell lines (H1650, SPC-A1, Calu3, A549 and H1299) than in the normal lung cell line (Fig. 1c). A549 and H1299 cells demonstrated the highest levels of MIAT expression, and H1650 cells demonstrated the lowest expression level. Therefore, A549 and H1299 cells were selected for MIAT silencing, and H1650 cells were chosen for MIAT overexpression. We next analyzed the relationship of MIAT expression with several clinicopathological features of 80 NSCLC patients. Increased MIAT expression was significantly associated with advanced pathological stage (Fig. 1d). Moreover, Kaplan-Meier and log-rank test revealed that high MIAT expression was significantly correlated with poor prognosis (Fig. 1e).

### MIAT promotes growth, invasion, and migration of NSCLC cells in vitro

**Fig. 2:**
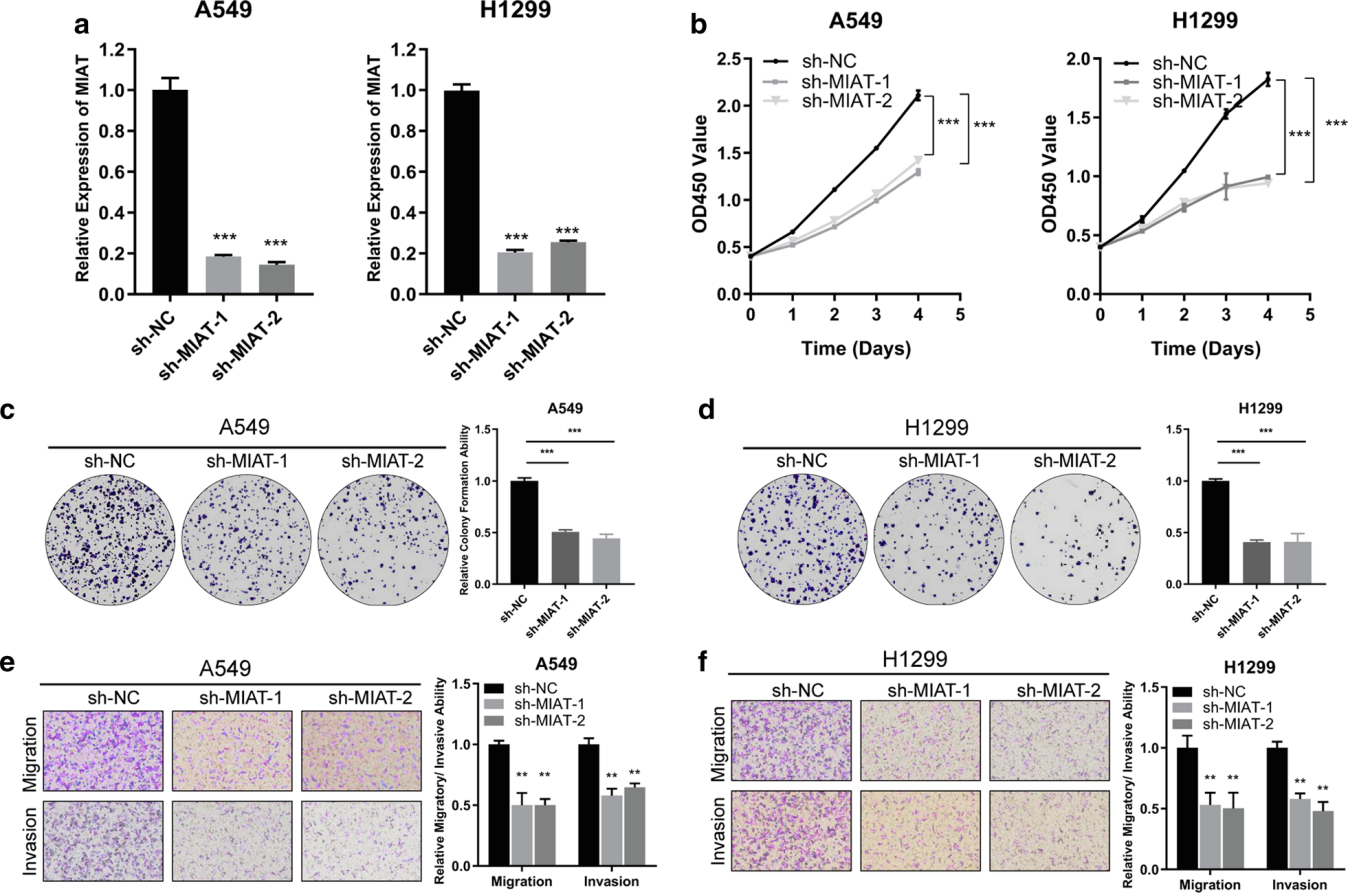
MIAT acts as an oncogene in NSCLC cells. **a** qRT-PCR analysis of MIAT expression in A549 and H1299 cells treated with two shRNAs. **b**–**f** Cell proliferative, migratory and invasive capabilities were assessed by CCK-8 assay (**b**), colony formation assay (**c**, **d**), and transwell assays (**e**, **f**) after knocking down MIAT in A549 and H1299 cells. **p < 0.01, ***p < 0.001

**Fig. 3:**
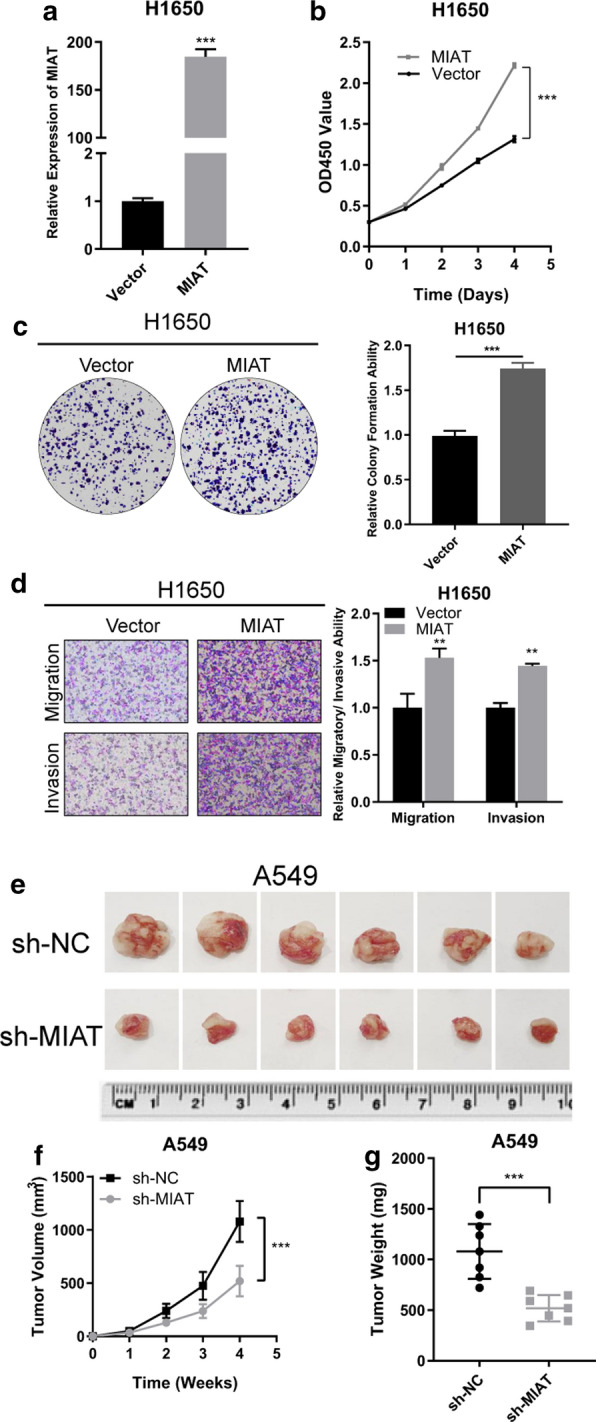
**a** qRT-PCR analysis of MIAT expression in H1650 cells stably overexpressing MIAT. **b**–**d** Cell proliferative, migratory and invasive capabilities were assessed by CCK-8 (B), colony formation assay (**c**) and transwell assay (**d**) after overexpressing MIAT in H1650 cells. **e** Representation picture of the A549 xenograft tumor. **f**, **g** Tumor volumes and weights were significantly deceased after MIAT silencing. **p < 0.01, ***p < 0.001

To assess the influence of MIAT on the malignant behaviors of NSCLC cells, loss and gain-of-function assays were conducted. The effect of knockdown or overexpression of MIAT was measured by qRT-PCR. After transfecting A549 and H1299 with the shRNAs, MIAT was successfully knocked down (Fig. 2a). CCK-8 and colony formation assays showed that knockdown of MIAT suppressed cell proliferation (Fig. 2b) and colony-forming abilities (Fig. 2c, d) in both A549 and H1299 cells in comparison with those in negative control cells. Additionally, transwell assays showed that invasion and migration were significantly inhibited after MIAT knockdown in both A549 and H1299 cells (Fig. 2e, f). Furthermore, a MIAT-overexpressing plasmid was constructed and successfully transfected into H1650 cells (Fig. 2a). Stable overexpression of MIAT in H1650 cells facilitated the cell proliferation, invasion and migration (Fig. 3b–d). To sum up, these assays revealed that MIAT promotes the proliferation, migration, and invasion of NSCLC cells in vitro.

### MIAT promotes NSCLC cell growth in vivo

To evaluate the influence of MIAT knockdown on tumor growth in vivo, A549 cells transfected with sh-MIAT or negative control cells were subcutaneously injected into nude mice. After injection, the tumor volumes were measured every week (Fig. 3e). The mean tumor volume (Fig. 3f) and weight (Fig. 3g) were significantly reduced in the MIAT knockdown group compared with those in the negative control group.

### MIAT binds directly to miR-149-5p in NSCLC cells

**Fig. 4:**
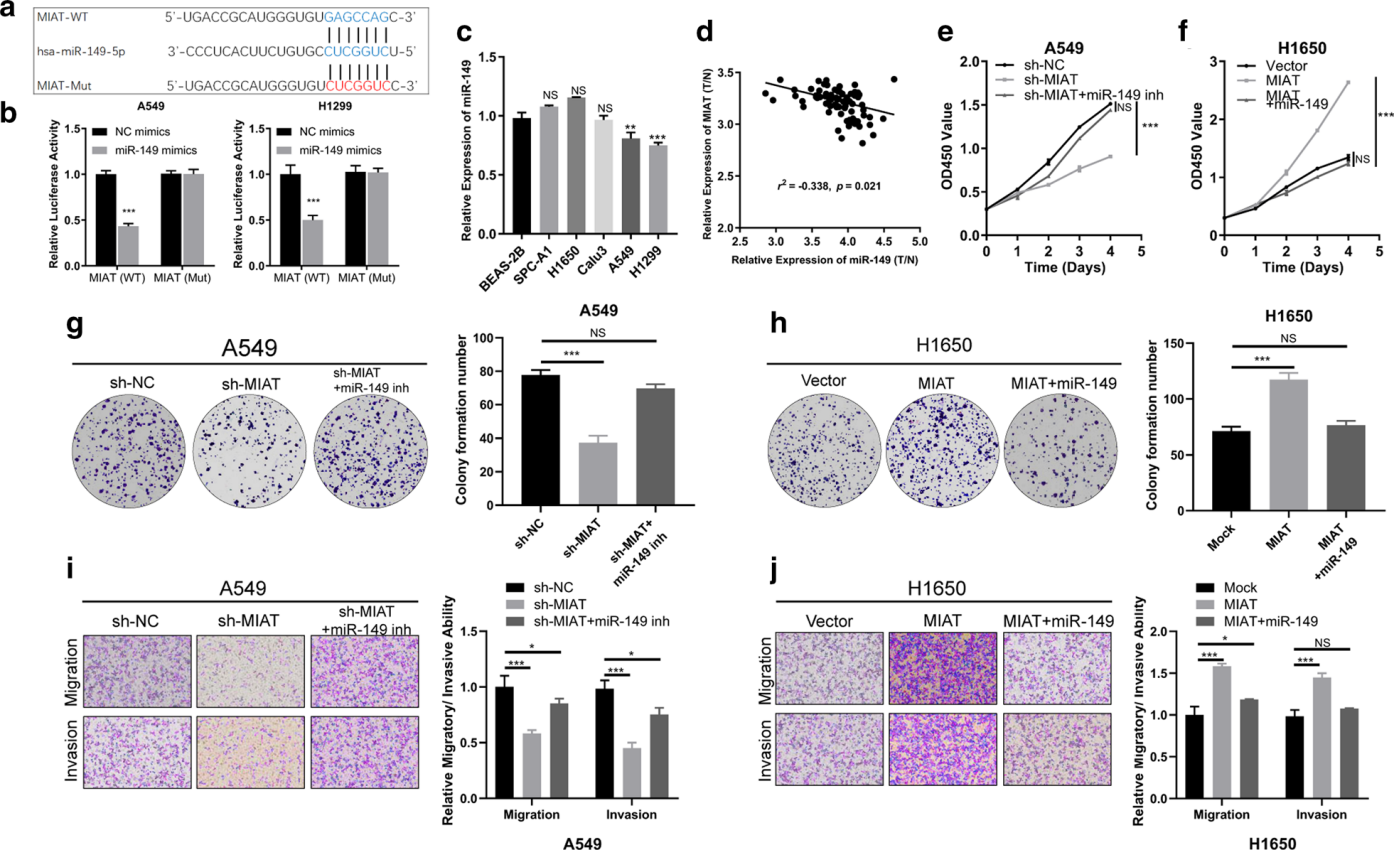
MIAT could serve as a miR-149-5p sponge, and miR-149-5p counters the oncogenic effects of MIAT in NSCLC cells. **a** Schematic representation of the potential binding sites of miR-149-5p and MIAT. **b** The luciferase activities were assessed after wild-type or mutant MIAT luciferase reporter vector was co-transfected with miR-149-5p mimic or negative control mimic into A549 and H1299 cells. **c** The expression levels of miR-149-5p in lung cancer cells and normal lung cells were measured by qRT-PCR. **d** Pearson correlation analysis showed that miR-149-5p expression was negatively correlated with that of MIAT. **e**, **g**, **i** CCK-8, Colony formation and transwell assays revealed that silencing of MIAT inhibited A549 and H1299 cell proliferation, and these inhibition effects were attenuated by miR-149-5p inhibitor. **f**, **h**, **j** Overexpression of MIAT promoted A549 and H1299 cell proliferation, and these inhibition effects were reversed by miR-149-5p mimic. *p < 0.05, ***p < 0.001

It has been reported that lncRNAs mainly function as sponges of miRNAs in regulation of gene expression [15]. Bioinformatics analysis revealed only miR-149-5p as a target in both DIANA LncBase and TargetScan databases; therefore, it was selected as the best potential target of MIAT (Fig. 4a). The interaction between MIAT and miR-149-5p was confirmed by dual-luciferase reporter assay, showing that miR-149-5p mimic clearly reduced the luciferase activity of wild-type MIAT in comparison with that of the negative control. Meanwhile, the miR-149-5p mimic had no influence on the luciferase activity of MIAT-mut (Fig. 4b). The results of qRT-PCR showed that miR-149-5p was significantly underexpressed in A549 and H1299 cells (Fig. 4c). Pearson correlation analysis showed that miR-149-5p expression was negatively correlated with that of MIAT (Fig. 4d). These results demonstrated that MIAT could directly bind to miR-149-5p.

### miR-149-5p reverses the oncogenic roles of MIAT in NSCLC cells

To explore the effects of MIAT and miR-149-5p in NSCLC progression, rescue assays were conducted to measure how the MIAT/miR-149-5p axis affects the proliferative, migratory, and invasive abilities of NSCLC cells. The results of CCK-8 and Colony formation assays revealed that silencing of MIAT inhibited A549 and H1299 cell proliferation, and these inhibition effects were attenuated by miR-149-5p inhibitor (Fig. 4e, g). Transwell assays showed that MIAT knockdown remarkably attenuated the migratory and invasive abilities, and the effects could be counteracted by miR-149-5p inhibitors (Fig. 4i). On the contrary, MIAT overexpression promoted H1650 cell proliferation, migration, and invasion, and miR-149-5p weakened this promotion (Fig. 4f, h and j).

### FOXM1 was a direct target gene of miR-149-5p

**Fig. 5:**
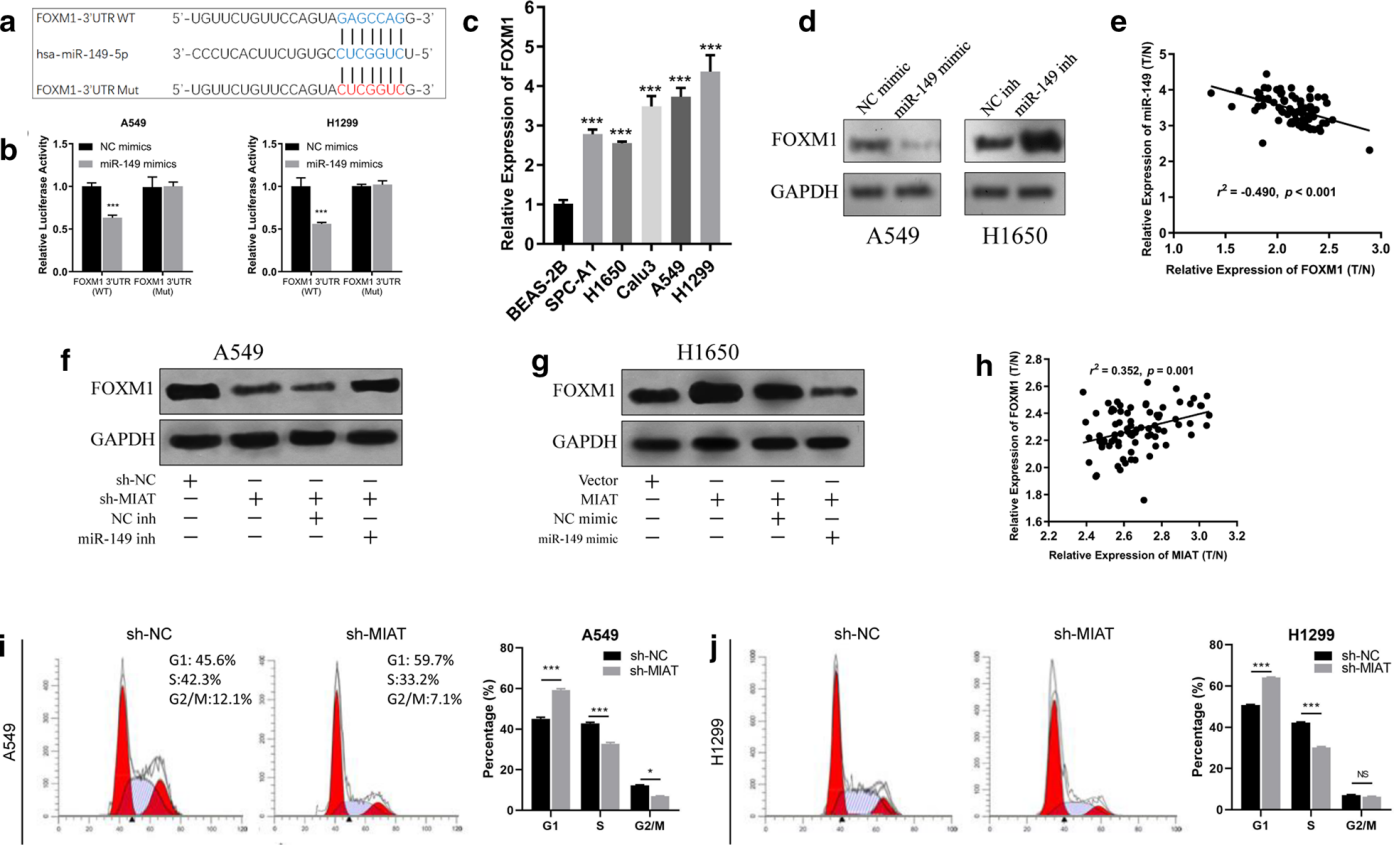
FOXM1 was the direct target of miR-159-5p. **a** Schematic representation of the potential binding sites of miR-149-5p with MIAT and FOXM1. **b** The luciferase activities were assessed after wild-type or mutant FOXM1 3′UTR luciferase reporter vector was co-transfected with miR-149-5p mimic or negative control mimic into A549 and H1299 cells. **c** The expression levels of FOXM1 in lung cancer cells and normal lung cells were measured by qRT-PCR. **d** Western blot assay shows FOXM1 expression in A549 cells and H1650 cells after transfected with miR-149-5p mimic or inhibitor. **e** FOXM1 was negatively correlated with miR-149-5p expression in NSCLC tissues. **f** Knockdown of MIAT decreased FOXM1 expression in A549 cells, and this effect was reversed by co-transfection of miR-149-5p inhibitor. **g** Overexpression of MIAT increased FOXM1 expression in H1650 cells, and this effect was reversed by co-transfection of miR-149-5p mimic. **h** FOXM1 was positively correlated with MIAT expression in NSCLC tissues. **i**, **j** Flow cytometric analysis of the cell cycle after MIAT knockdown in A549 and H1299 cells. ***p < 0.001

Targetscan was used to predict the target genes of miR-149-5p, and the FOXM1 gene was revealed to be the best potential candidate (Fig. 5a). We observed that co-transfection of miR-149-5p mimic and reporter plasmids markedly attenuated the luciferase activity. Oppositely, co-transfection of miR-149-5p mimic and mutated vectors demonstrated no influence on luciferase activity (Fig. 5b). The results of qRT-PCR showed that miR-149-5p was significantly overexpressed in all lung cancer cell lines, especially in A549 and H1299 cells (Fig. 5c). Moreover, western blotting revealed that miR-149-5p inhibitors significantly increased FOXM1 expression in A549 cells, whereas miR-149-5p mimic significantly decreased FOXM1 expression in H1650 cells (Fig. 5d). Pearson correlation analysis demonstrated that FOXM1 expression was negatively correlated with that of miR-149-5p as determined by qRT-PCR in NSCLC tissues (Fig. 5e). These findings confirmed that FOXM1 was indeed a direct target of miR-149.

### MIAT sponged miR-149-5p to upregulate FOXM1

To investigate whether MIAT sponges miR-149-5p to regulate FOXM1 expression, we determined FOXM1 expression using western blotting. Silencing MIAT attenuated FOXM1 expression, and this could be reversed by co-transfection of miR-149-5p inhibitor (Fig. 5f). Additionally, MIAT overexpression significantly augmented FOXM1 expression, whereas miR-149-5p mimic reversed this effect (Fig. 5g). Moreover, FOXM1 was positively correlated with MIAT expression in NSCLC tumor tissues (Fig. 5h).

### MIAT knockdown led to G1/S arrest

To determine the whether FOXM1 inhibition caused by MIAT knockdown could affect cell cycle, the cell cycle arrest analysis was performed. The results showed that the percentage of cells in the G1 phase was significantly increased in sh-MIAT-transfected A549 and H1299 cells in comparison with that in sh-NC cells. Besides, the percentages of cells in the S phase were markedly deceased after MIAT knockdown. Therefore, knockdown of MIAT inhibited the expression of FOXM1, and subsequently suppressed the G1/S transition of NSCLC cells (Fig. 5i, j).

## Discussion

**Fig. 6:**
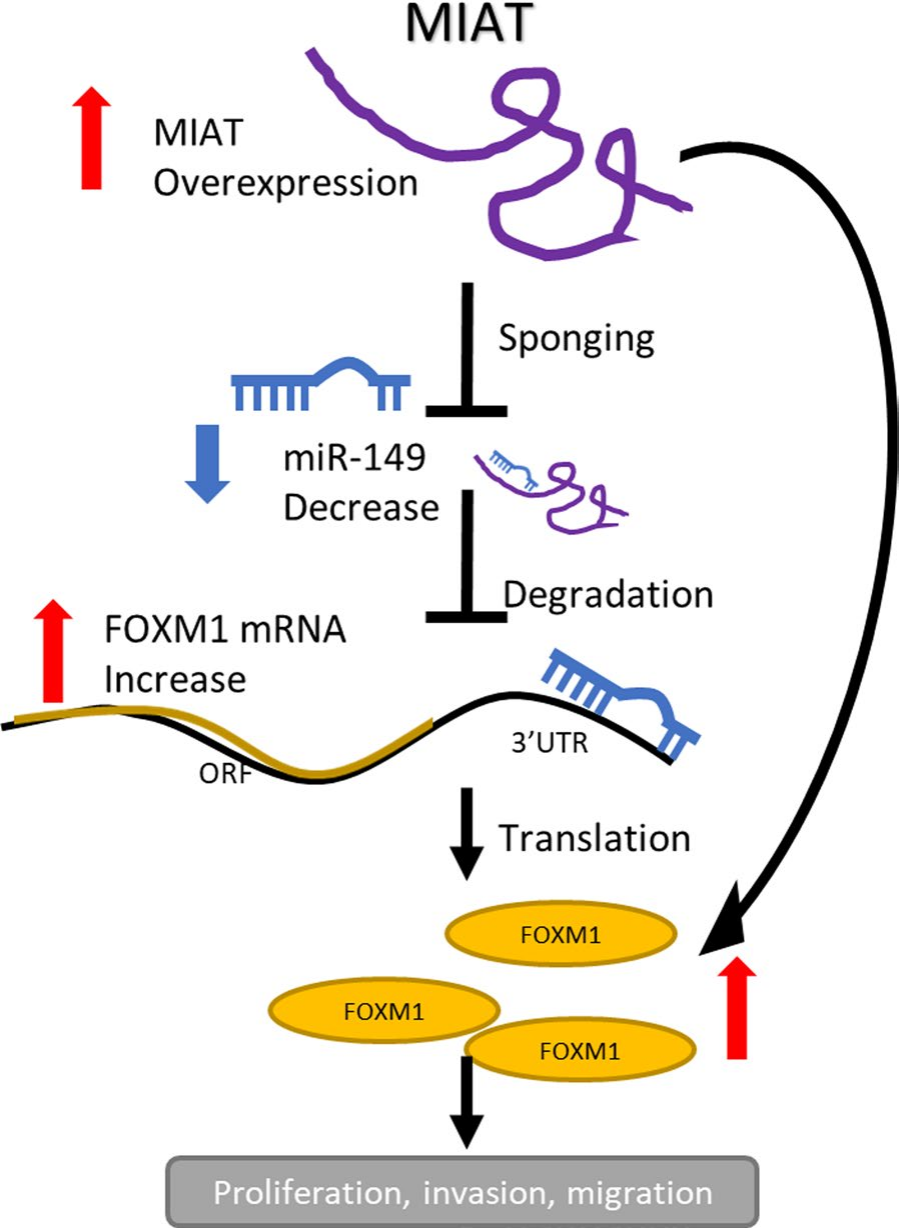
Schematic illustration of the MIAT/miR-149/FOXM1 regulatory axis

In the present study, we demonstrated that MIAT might serve as an oncogene in NSCLC. MIAT expression was significantly increased in NSCLC compared with that in adjacent normal tissues. The increase of MIAT expression was also observed in NSCLC cell lines. MIAT was significantly increased in A549 and H1299 cells, while H1650 cells demonstrated a relatively lower expression level. This difference may be attributed to the origin of the cell lines. H1650 cells derived from minimally invasive lung adenocarcinoma, therefore, it may be less invasive than A549 and H1299. We found that high expression of MIAT was correlated with advanced TNM stage. More importantly, patients with increased MIAT expression had a considerably worse survival than those with low MIAT expression. Subsequently we validated the biological functions of MIAT in NSCLC cells. Our data showed that MIAT promoted NSCLC cell proliferation, invasion, and migration in vitro and in vivo, whereas knockdown of MIAT reversed these effects, suggesting that MIAT might act as an oncogene in NSCLC. Mechanistically, MIAT could up-regulate FOXM1 expression by sponging miR-149-5p. Taken together, we identified a novel MIAT/miR-149-5p/FOXM1 regulatory axis that promotes NSCLC progression (Fig. 6).

Competitive endogenous RNA (ceRNA) is a common mechanism of lncRNAs, among which lncRNAs regulate targeted mRNAs through sponging certain miRNAs. A recent study showed that MIAT acted as a sponge to positively modulate the expression of anti-phagocytic molecule CD47 through sponging miR-149-5p [16]. Wang et al. revealed that MIAT promoted cell viability, proliferation, migration, and the EMT in renal interstitial fibrosis via regulation of the miR-145/EIF5A2 axis [17].

In the present study, we selected several potential target miRNAs by bioinformatics methods, and the predicted target miR-149-5p was confirmed to directly bind to MIAT using dual-luciferase reporter assay. Additionally, the results showed that FOXM1, a well-known cancer related gene, is the direct target of the MIAT/miR-149-5p axis.

Aberrant expression of miR-149-5p has been investigated in multiple types of cancers. Previous reports have indicated that miR-149-5p functions as a tumor suppressor, inhibiting proliferation, cell cycle progression, invasion, and metastasis, and it is downregulated in non-small cell lung cancer [18], glioma [19], breast cancer [20], and colorectal cancer [21]. FOXM1 is a member of the FOX family of transcription factors regulating the expression of cell cycle genes essential for DNA replication and mitosis [22–24]. FOXM1 plays an important role in the control of cell proliferation and is considered as a human proto-oncogene [25]. Abnormal upregulation of FOXM1 is involved in the oncogenesis of the majority of solid human cancers [26–29]. Xu et al. reported that FOXM1 is the direct target of miR-149-5p and that the latter attenuated the invasive and migratory ability of colorectal cancer [21]. Luo et al. observed a negative correlation between miR-149-5p and FOXM1 mRNA levels, and suggested that miR-149-5p inhibited FOXM1 expression [30]. Zhang et al. indicated that MIAT promoted esophageal squamous cell carcinoma progression via targeting INCENP/miR-1301-3p axis and interacting with SOX2. In our study, co-transfection of sh-MIAT and miR-149-5p inhibitor could increase cell viability when compared with the transfection of sh-MIAT alone. We think it is because that MIAT increased cell viability by sponging miR-149-5p, but transfection of sh-MIAT alone decreased the sponging effect, and more miR-149-5p was liberated and could exert suppressive effect on cell viability. After co-transfection of miR-149-5p inhibitor, the suppressive effect of miR-149-5p was countered, thus the cell viability increased. Besides, MIAT augmented FOXM1 expression, whereas the effect could be countered by co-transfection with miR-149-5p. MIAT silencing repressed FOXM1 expression, but the effects could be abolished after miR-149-5p inhibitor co-transfection. FOXM1 is the direct target of miR-149-5p, while MIAT acts as a sponge of miR-149-5p to upregulate FOXM1 expression in NSCLC cells.

## Conclusions

In summary, we have identified a novel circRNA, MIAT, which is upregulated in NSCLC tissues and cell lines, and a high MIAT expression is associated with a poor prognosis in NSCLC patients. MIAT functions as an oncogene, enhancing the proliferation, migration, and invasion of NSCLC by sponging miR-149-5p to upregulate FOXM1. We demonstrated a novel MIAT/miR-149/FOXM1 regulatory axis in NSCLC, which may serve as a diagnostic biomarker and therapeutic target for the treatment of NSCLC.

## Data Availability

Not applicable.
